# Extremophilic Fungi from Marine Environments: Underexplored Sources of Antitumor, Anti-Infective and Other Biologically Active Agents

**DOI:** 10.3390/md20010062

**Published:** 2022-01-10

**Authors:** Lesley-Ann Giddings, David J. Newman

**Affiliations:** 1 Department of Chemistry, Smith College, 100 Green St., Northampton, MA 01063, USA; 2 NIH Special Volunteer, Wayne, PA 19087, USA; djnewman664@verizon.net

**Keywords:** extreme environments, marine fungi, bioactive compounds, marine natural products, fungal cultivation strategies

## Abstract

Marine environments are underexplored terrains containing fungi that produce a diversity of natural products given unique environmental pressures and nutrients. While bacteria are commonly the most studied microorganism for natural products in the marine world, marine fungi are also abundant but remain an untapped source of bioactive metabolites. Given that their terrestrial counterparts have been a source of many blockbuster antitumor agents and anti-infectives, including camptothecin, the penicillins, and cyclosporin A, marine fungi also have the potential to produce new chemical scaffolds as leads to potential drugs. Fungi are more phylogenetically diverse than bacteria and have larger genomes that contain many silent biosynthetic gene clusters involved in making bioactive compounds. However, less than 5% of all known fungi have been cultivated under standard laboratory conditions. While the number of reported natural products from marine fungi is steadily increasing, their number is still significantly lower compared to those reported from their bacterial counterparts. Herein, we discuss many varied cytotoxic and anti-infective fungal metabolites isolated from extreme marine environments, including symbiotic associations as well as extreme pressures, temperatures, salinity, and light. We also discuss cultivation strategies that can be used to produce new bioactive metabolites or increase their production. This review presents a large number of reported structures though, at times, only a few of a large number of related structures are shown.

## 1. Introduction

Natural products have been a rich source of therapeutics since the beginning of civilization. To date, ~60% of all small-molecule drugs approved by the US Food and Drug Administration (FDA), or their equivalent in other countries, have been inspired by these secondary metabolites [1]. Many are anti-infectives and antitumor agents that have been isolated from microbes, including those with symbiotic associations. While microbes are a prolific source of bioactive molecules used to treat cancer and infectious diseases, chemists have been repetitively rediscovering natural products, thus leading to the requirement for a system for the rapid dereplication of known compounds and/or their close relatives. Microbes usually express fewer biosynthetic gene clusters (BGCs) when grown under standard laboratory conditions, even though they have the genetic potential to express more secondary metabolic BGCs. To circumvent these limitations, scientists are either cultivating microbes under nominally non-standard laboratory conditions to activate the expression of cryptic BGCs [2], using genetic engineering and ‘omics methods to identify and directly express BGCs [3], or they are isolating microbes from understudied environments [4].

Extreme ecosystems represent new frontiers for drug discovery, as these locations contain unique environmental variables that are not found in mesophilic locales and influence microbial metabolism. Extreme environments have been underexplored due to the challenges in accessing these locales. However, more sampling tools and techniques have been developed to study marine environments, presenting new opportunities for exploration. The marine world is vast, covering ~70% of Earth, including some of the most extreme ecosystems on the planet. Extreme marine environments are characterized as having high pressures, and temperatures that can range from close to freezing to over 250 °C at the “black smokers”, severe ultraviolet radiation, high salt or metal concentrations, symbiosis, or more than one combination of these variables. Microbes constitute much of the biomass in the sea and are prominent producers of secondary metabolites, with a wide range of potent bioactivities against tumor cells and other pathogens, plus potential in other diseases. Thus, bioprospecting these environments for new drugs is an attractive prospect, especially as marine natural products have high hit rates in a broad range of bioassays, with many in clinical and preclinical trials. For a current assessment, the web site, www.marinepharmacology.org (accessed on 12 December 2021), is kept up to date, covering approved drugs from marine sources and compounds that are in clinical trials from Phase I to Phase III plus selected preclinical candidates. Most of the information is from the NIH trials database (www.clinicaltrials.gov accessed on 12 December 2021), but it also includes data from comparable databases from other countries.

Most microbe-derived marine natural products tend to be isolated from bacteria, which make up much of the biomass in seawater (i.e., >10^5^ cells per milliliter) and are extremely well represented in deep oceanic “muds”. However, fungi are also abundant, as 10^3^ to 10^4^ fungal cells are contained in a milliliter of seawater. Approximately 38% of the reported ~23,000 bioactive microbial metabolites are of fungal origin and only 5% of the microbial taxa had been identified as of 2008, 10% of which are fungi [5]. Fungi have larger genomes with more BGCs, resulting in greater chemodiversity [6,7]. Furthermore, they have produced numerous clinically approved anti-infectives and antitumor drugs, as mentioned earlier, but remain understudied.

Why are there disparities in the isolation of marine natural products from fungi? While genetic engineering and ‘omics methods are being used to isolate an increasing amount of bacterial natural products, these methods are still in their infancy insofar as fungi are concerned. Cultivation which is still one of the main ways to discover bioactive metabolites continues to be a challenge when working with marine fungi. Scientists are still using antiquated methods that promote the growth of generalist genera, such as *Penicillium* and *Aspergillus* to culture marine fungi [8]. In 2019, we published a compendium of bioactive compounds isolated from marine fungi [9]. Herein, we update that list, focusing on anti-infective and antitumor agents as well as other fungal natural products with potential from marine environments. This review aims to highlight the bioactive metabolites found from culturable fungi isolated from marine environments and cultivation methods that have been found to increase the production of diverse bioactive metabolites.

Although we are listing significant numbers of compounds (approximately 200) in this review, we will only show the structures of compounds for which biological activities have been reported and will give the names of the others with suitable references, so that readers may consult the original literature for related compounds without a biological activity reported at that time.

### 1.1. Antitumor Agents from Deep-Sea Sediments

It should be mentioned at this point that it was well known in the early days of antimicrobial assays, though never formally reported from the pharmaceutical industry, that compounds with antitumor activities in vitro also inhibited Gram-positive bacteria at comparable concentrations in disc diffusion assays (DJN Personal Observations). The following examples will be arranged as best we can, by the area(s) from which the sediments were collected for subsequent isolation of fungi.

### 1.2. Indian Ocean

Numerous potential antitumor agents (some also with antibacterial activity) continue to be isolated and reported from deep-sea-derived fungi collected from the Indian Ocean. The new diterpenoids, longidiacids A (**1**) and B, polyketides, and the cytochalasin analogs, longichalasins A and B (**2**), were isolated from the fungus *Diaporthe longicolla* FS429 [10] obtained from deep-sea sediment collected at a depth of 3000 m. Longidiacid A (**1**) and longichalasin B (**2**) inhibited tyrosine phosphatase B in *Mycobacterium tuberculosis* cells by 35.4% and 53.5%, respectively, and longichalasin B (**2**) exhibited antiproliferative activity against glioblastoma cells (SF-268) with an IC_50_ value of 16.44 μM. 

From sediment collected at a depth of 3471 m, the fungus *Cladosporium cladosporiodes* HDN14-342 yielded the polyketides, clindanones A and B as well as cladosporols F and G (**3**, **4**) following cultivation [11]. Cladosporols F and G exhibited cytotoxic activities against the human cervical (HeLa), leukemia (K562), and colon (HCT-116) cancer cells with IC_50_ values ranging from 3.9 to 23.0 μM depending upon the particular cell line. In 2017, Li et al. published revisions of the initial structures of these two metabolites [12]. 

*Phomopsis lithocarpus* FS508 was isolated from a sediment sample collected at 3606 m and fermented, producing several benzophenone aldehydes, including tenellone H (**5**). This benzophenone exhibited cytotoxic activity against liver (HepG-2; IC_50_ 16 μM) and lung (A549; IC_50_ 17.6 μM) cancer cell lines [13]. At the even deeper depth of 5752 m, the trimeric peniphenylanes A and B and dimeric peniphenylanes C–G were reported from *Penicillium fellutanum* HDN14-323 isolated from that deep-sea sediment. Of these seven compounds, only peniphenylane D (**6**) exhibited reasonable cytotoxic activity against the cervical cancer cell (HeLa; IC_50_ = 9.3 μM) [14]. 

### 1.3. Seas near China

Moving further East, several cytotoxic agents have been reported from fungi from deep-sea sediment collected in the South China Sea. For example, the cytotoxic agent acaromycin A (**7**) along with acaromyester A were isolated from *Acaromyces ingoilii* FS121 collected from deep-sea sediment at a depth of 3415 m. The naptha-[2,3-b] pyrandione analog acaromycin A (**7**) exhibited cytotoxic activities against human breast (MCF-7), brain (SF-268), liver (HepG-2), and lung (NCI-H460) cancer cells with IC_50_ values less than 10 μM [15].

At a deeper depth of 3739 m, engyodontiumones A–J and 2-methoxyl-cordyol C were isolated from *Engyodontium album* DFFSCS02. Engyodontiumone H (**8**) exhibited cytotoxic activity against human histiocytic lymphoma U937 cells (IC_50_ = 4.9 μM) together with antibacterial activity against *Escherichia coli* and *Bacillus subtilis* at a concentration of 25 μg/disc (which is low activity for a disc diffusion assay). Engyodontiumin A (**9**), isolated from the same fungus, exhibited antimicrobial activities against *Aspergillus niger*, multidrug-resistant *Staphylococcus aureus*, *Vibrio vulnificus*, *V. rotiferianus*, and *V. campbellii* [16].

### 1.4. Eastern Pacific Ocean

Several new breviane spiroditerpenoids, breviones F–I, were isolated from a deep ocean sediment-derived *Penicillium* sp. (MCCC 3A00005) collected in the East Pacific Ocean at a depth of 5115 m [17]. Brevione F (**10**) inhibited HIV-1 replication in C8166 cells with an EC_50_ value of 14.7 μM. Breviones F–H (**10**–**12**) exhibited minimal cytotoxic activity against HeLa cells (25 to 50% growth inhibition at 10 μg/mL). Finally, from these agents, brevione I (**13**) was cytotoxic against the breast cancer line MCF-7 (IC_50_ = 7.44 μM) but barely active against the lung cancer A549 cell line (IC_50_ = 32.5 μM) [18].

**Figure 1:**
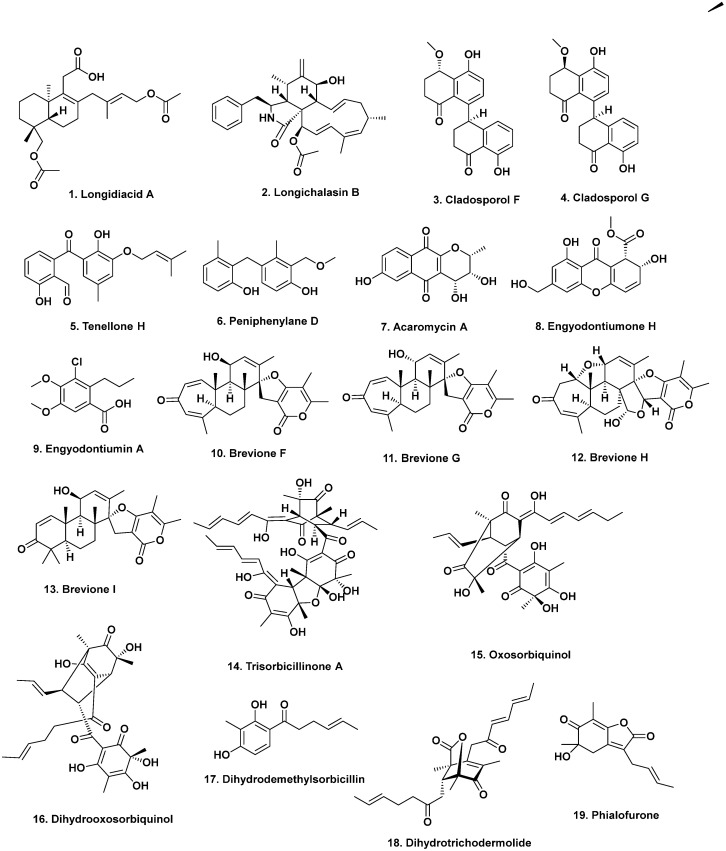
Antitumor Active Agents {Multiple Ocean Areas} (Structures **1** to **19**).

A series of sorbicillinoid derivatives, trisorbicillinone A–D, oxosorbiquinol, dihydrooxosorbiquinol, dihydrodemethylsorbicillin, dihydrotrichodermolide, and phialofurone, were isolated from the deep ocean fungus *Phialocephala* sp. Fl30 r collected from sediment at 5059 m in the East Pacific Ocean [19,20,21]. Of these agents, only trisorbicillinone A (**14**) exhibited any significant cytotoxic activity against the HL60 tumor cell line (IC_50_ = 3.14 μM) [19]. Oxosorbiquinol (**15**) and its dihydro derivative (**16**) were marginally to effectively inactive as cytotoxins against the following tumor cell lines; leukemia (P388, HL60, and K562) and the hepatocellular BEL7402 cell lines with IC_50_ values from 8.9 to 68.2 μM, respectively [19]. By contrast, dihydrodemethylsorbicillin (**17**), dihydrotrichodermolide (**18**), and phialofurone (**19**, Figure 1) exhibited cytotoxic activity against the leukemia cell lines (P388 and K562) with IC_50_ values ranging from 0.1 to 22.9 μM [21].

## 2. Anti-Infective Agents from Deep-Sea Fungi

### 2.1. South Atlantic Ocean

**Figure 2:**
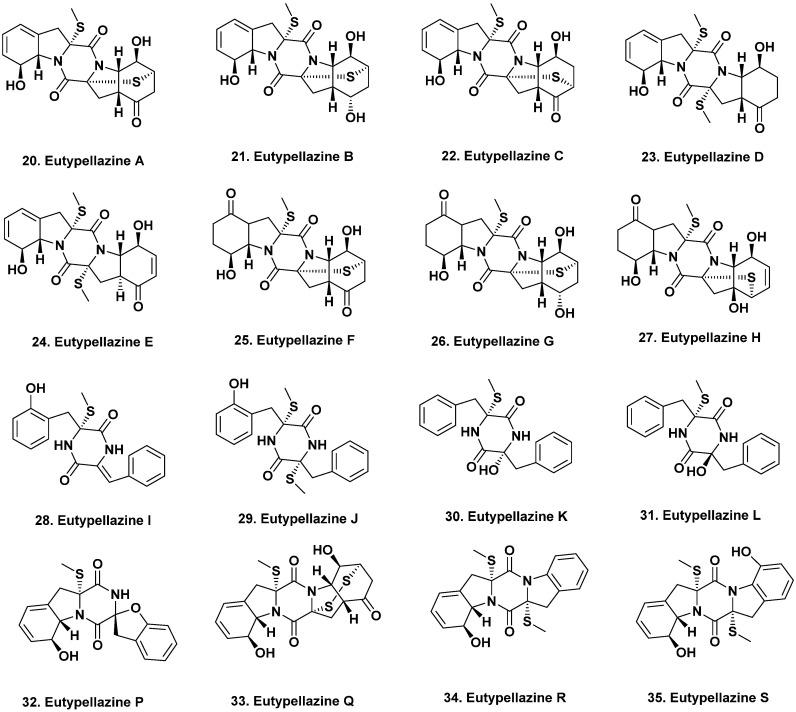
Anti-HIV and Antibacterial Active Agents {South Atlantic Ocean, South China Sea, Indian and West Pacific Oceans} (Structures **20** to **35**).

Marine fungi are also abundant sources of anti-infectives, including potential antifungal, antibacterial, antiprotozoal, and antiviral agents. For example, 19 thiodiketopiperazine alkaloids, eutypellazines A–S, were isolated from the marine-derived fungus *Eutypella* sp. MCCC 3A00281 collected at a depth of 5610 m in the South Atlantic Ocean [22,23]. Eutypellazines A–L (**20**–**31**) exhibited anti-HIV activity against pNL4.3Env-Luc co-transfected 293T cells with IC_50_ values ranging from 3.2 to 18.2 μM. Eutypellazines P–S (**32**–**35**, Figure 2) inhibited the growth of *S. aureus* ATCC 25923 and vancomycin-resistant *Enterococci* with MIC values ranging from 16 to 32 μM [22,23].

### 2.2. South China Sea

The alkaloids arthpyrones D–K were isolated from *Arthrinium* sp. UJNMF0008 collected from the South China Sea at a depth of 3858 m. Of these eight, four, arthpyrones F–I (**36**–**39**), exhibited antibacterial activity against *Mycobacterium smegmatis* and *S. aureus* with IC_50_ values ranging from 1.66 to 42.8 μM [24]. From the deep-sea fungus, *P. brevicompactum* DFFSCS025, isolated from sediment collected at 3928 m depth in the South China Sea, two alkaloids, breviamides X and Y, and two mycochromenic acid derivatives, 6-(methyl 3-methylbutanoate)-7-hydroxy-5-methoxy-4-methylphthalan-1-one (**40**) and (3′*S*)-(*E*)-7-hydroxy-5-methoxy-4-methyl-6-(2-(2-methyl-5-oxotetrahydrofuran-2-yl)vinyl) isobenzofuran-1(3H)-one, were purified. Of these four, only compound **40** exhibited significant antifouling activity against the bryozoan *Bugula neritina* with an EC_50_ value of 13.7 μM and an LC_50_/EC_50_ value > 100 [25].

A small series of compounds were isolated from fermentation of the fungus *Emericella* sp. SCSIO 05240, collected from sediment at a depth of 3258 m in the South China sea. These were four new prenylxanthones, named as emerixanthones A–D. These four agents structurally differ by chlorination, methylation, and hydroxylation, and of the four congeners, two, emerixanthones A (**41**) and C (**42**), exhibited weak antibacterial activity against several bacterial pathogens, including *E. coli*, *Klebsiella pneumonia*, *S. aureus*, *E. faecalis*, *Acinetobacter baumanni*, and *Aeromonas hydrophilia*. Interestingly, one of the remaining two compounds, emerixanthone D (**43**), exhibited mild antifungal activity against several agricultural pathogens, including *Fusarium* sp., *Penicillium* sp., *A. niger*, *Rhizoctonia solani*, *Fusarium oxysporium f.* sp. *niveum*, and *F. oxysporium f.* sp. *cucumeris* [26].

### 2.3. Indian Ocean

From sediment collected in the Indian Ocean at a depth of 3972 m, the versicoloids A and B as well as two 4-aryl-quinolin-2-one alkaloids and prenylated xanthones, versicones A–D, were isolated from culturing the fungus *A. versicolor* SCSIO 05879. Of these four compounds, only versicoloids A and B (**44**, **45**) demonstrated activity against the plant pathogen *Colletotrichum acutatum* (MIC 1.6 μg/mL) [27].

### 2.4. West Pacific Ocean

**Figure 3:**
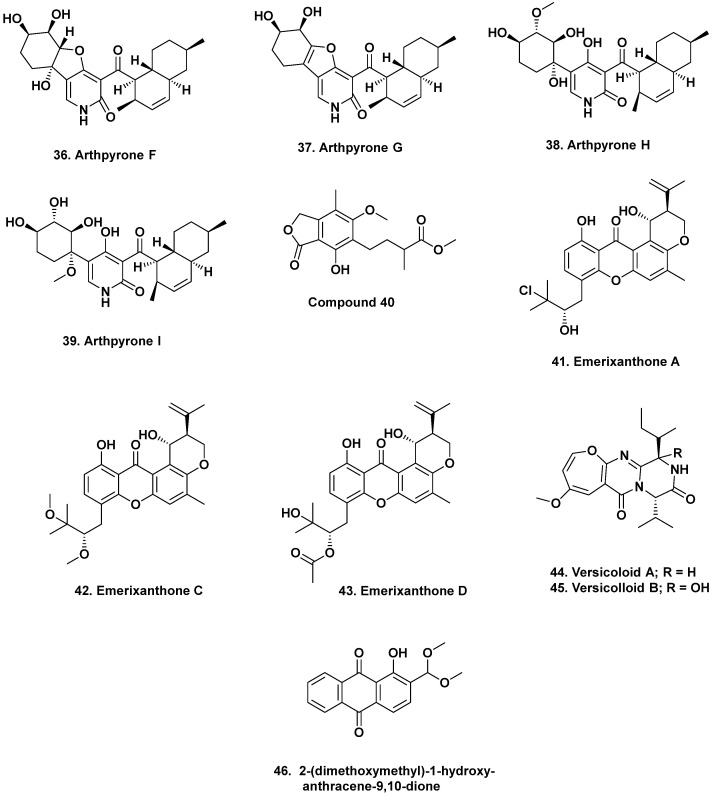
Biologically Active Agents, Indian and West Pacific Ocean (Structures **36** to **46**).

From sediment collected at a depth of 2869 m in the West Pacific Ocean, the fungus *A. versicolor* was isolated and upon fermentation produced the anthraquinone antimicrobial agent 2-(dimethoxymethyl)-1-hydroxyanthracene-9,10-dione (**46**, Figure 3). This compound exhibited antibacterial activity against multidrug-resistant strains of *S. aureus* ATCC 43300 (MIC 3.9 μg/mL) and CGMCC 1.12409 (MIC 7.8 μg/mL). In addition, it was also weakly active against strains of *Vibrio* (MICs of 15.6–62.5 μg/mL). Molecular docking studies with topoisomerase IV and AmpC β-lactamase further supported its antibacterial properties [28].

## 3. Other Biologically Active Agents from Deep-Sea Fungi

### 3.1. (Probably) South China Sea

Anti-inflammatory agents have also been isolated from extremophilic marine fungi. For example, the diketopiperazine alkaloids brevicompanines D–H were isolated from a deep ocean-sediment isolate of *Penicillium* sp. collected at a depth of 5080 m. Of these five compounds, only brevicompanines E (**47**) and H (**48**) inhibited lipopolysaccharide-induced nitrous oxide production in microglial cells [29].

### 3.2. Antarctica

**Figure 4:**
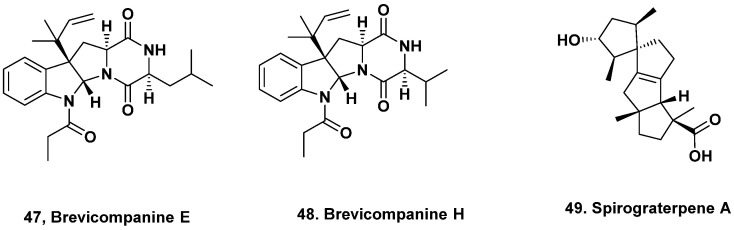
Biologically Active Agents {probably) South China Sea and Antarctica} (Structures **47** to **49**).

Another strain of *Penicillium, P. granulatum* MCCC 3A00475, isolated from deep-sea sediment collected at a depth of 2284 m from Prydz Bay in Antarctica yielded the antiallergic spirotetracyclic diterpene, spirograterpene A (**49**, Figure 4). This compound demonstrated a weak antiallergic activity on immunoglobulin E-mediated rat mast RBL-2H3 cells [30].

## 4. Bioactive Compounds from Fungal Endophytes of Mangroves

Marine fungi are often involved in mutualistic interactions, as their survival may well require relationships including symbiosis with other organisms. Many endophytic fungi have been isolated from mangroves, which are intertidal wetland environments comprising closely associated plants, animals, and microbes from many genera. These environments line (sub)tropical habitats, comprising one-quarter of the tropical coastline in the world (over 15.5 million hectares) [31].

Mangroves are frontiers between land and sea, a mixture of freshwater and saltwater, exporting plant detritus and faunal biomass to support life offshore. Currently, the Bandaranayake paper [31] has been cited over 350 times, demonstrating the “ecological value” of these marine/freshwater environments. These environments are complex and rich in fungal species diversity, largely based on the availability of colonizable substrata. Within these environments, fungi play an important role in the nutrient cycles, transforming polymeric substances into smaller and simpler organic matter that can be used by other organisms [32]. These secondary metabolic products help organisms cope with biotic stress factors, such as varying water and salt levels. There is also intense competition for space; thus, secondary metabolites are used as a form of chemical defense and/or offense to combat pathogens, herbivores, and other organisms.

Two recent reviews on anti-infectives from mangrove endophytic fungi were published by Deshmukh et al. in 2020 [33] and Cadamuro et al. in 2021 [34]. Many of these fungi have been reported from China, primarily due to their research groups now studying mangrove endophytes instead of their medicinal plant hosts [35]. These fungi typically belong to the genera *Aspergillus*, *Phomopsis*, *Pestalotiopsis*, and *Penicillium*, which is not surprising due to the cosmopolitan nature of these genera. The Chinese have a longstanding history of using traditional herbal mixtures to treat disease and have known about the toxic properties of marine natural products for centuries. However, many of these newly isolated compounds have been published in local journals or those dedicated to plant research, such as Phytochemistry, Planta Medica, Phytomedicine, or Phytochemical letters, and thus were not frequently read/cited by scientists involved in marine chemistry.

The She group at Sun Yat-Sen University has focused on identifying bioactive metabolites from mangrove-derived endophytic fungi from the South China Sea. Many of these metabolites have been published in Phytochemical Letters, Planta Medica, as well as Marine Drugs. For example, in 2014, Liu and coworkers published a paper in Planta Medica on three new vermistatin derivatives produced by a mangrove endophytic *Penicillium* sp. HN29-3B1 isolated from the sea mango *Cerbera manghas* in the South China Sea. With the exception of 5′-hydroxypenisimplicissin, both 6-demethylpenisimplicissin (**50**) and 2′-epihydroxydihydro-vermistatin (**51**) exhibited α-glucosidase inhibitory activity with IC_50_ values of 9.5 and 8.0 μM, respectively [36]. A year later, this group reported the production of pinazaphilones A and B, 4′-(*S*)-(3,5-dihydroxyphenyl)-4′-hydroxy-6′-methylcyclopent-1′-en-5′-one, 6′-methyl-[1,1′-biphenyl]-3,3′,4′,5-tetraol, and penicidone D from the same fungal isolate. Pinazaphilone B (**52**) and 6′-methyl-[1,1′-biphenyl]-3,3′,4′,5-tetraol (**53**) inhibited α-glucosidase with IC_50_ values of 28.0 and 2.2 μM, respectively, demonstrating their potential as antidiabetic agents [37].

The She group has reported several metabolites produced by endophytic *Talaromyces*. In 2011, they reported cytotoxic norsesquiterpene peroxides produced by an endophytic *T. flavus* isolated from the leaves of the mangrove plant *Sonneratia apetala* collected along the saltmarsh of the South China Sea [38]. Talaperoxides A to D (**54**–**57**), constituted two isomeric pairs exhibiting toxicity to brine shrimp at median lethal doses (LD_50_) of less than 10 ppm. These compounds were also evaluated for cytotoxicity against human breast (MCF-7 and MDA-MB-435), hepatoma (HepG2), cervical (HeLa), and prostate (PC-3) cancer cell lines. Talaperoxides B (**55**) and D (**57**) were cytotoxic against these cell lines with IC_50_ values ranging from 0.89 to 1.92 μg/mL. The group later reported two new benzophenone derivatives, peniphenone and methylpeniphenone, from the mycelia of an endophytic *Penicillium* sp. ZJ-SY2 isolated from the same plant [39]. Peniphenone (**58**) exhibited immunosuppressive activity against T-cell (concanavalin A-induced) and B-cell (liposaccharide-induced) proliferation in mouse splenic lymphocytes with IC_50_ values of 8.1 and 9.3 μg/mL, respectively. The methyl ester methylpeniphenone (**59**) exhibited weaker activity against T cells and B cells with IC_50_ values of 17.5 and 23.7 μg/mL, respectively. More recently, the group reported two new depsidones, talaromyones A and B, produced by cultures of *Talaromyces stipitatus* SK-4 isolated from the leaves of the mangrove plant *Acanthus ilicifolius* from the Shankou Mangrove Nature Reserve in China. The acetylated depsidone talaromyone B (**60**) exhibited antibacterial activity against *B. subtilis* (MIC = 12.5 μg/mL) as well as α-glucosidase activity (IC_50_ = 48.4 μM) [40].

Over the last decade, reports on the number of mangrove-derived strains of *Talaromyces* that produce bioactive metabolites have increased, especially as taxonomic revisions have been made to species with symmetrical biverticillate conidiophores. For more information on secondary metabolites from mangrove-associated strains of *Talaromyces*, the 2018 review by Nicoletti et al. in Marine Drugs is an excellent source of information [41].

Other research groups have reported bioactive compounds from fungal endophytes isolated from mangrove-associated medicinal plants. For example, Lin and coworkers published a paper in Phytochemistry describing four new polyketides produced by endophytic *Penicillium* sp. JP-1 isolated from the inner bark of *Aegiceras corniculatum* collected in Fujian, China [42]. *A. corniculatum* is a shrub known for its analgesic, cytotoxic, and antidiabetic properties [43,44]. Leptosphaerone C, penicillenone, arugosin I, and 9-demethyl FR-901235 isolated from fungal endophytes of this plant were evaluated for cytotoxic activity against human lung cancer (A-549) and murine lymphocytic leukemia (P388) cell lines. Leptosphaerone C (**61**) exhibited cytotoxic activity against human lung cancer cells (A-549; IC_50_ 1.45 μM) while penicillenone (**62**) was cytotoxic against murine lymphocytic leukemia (P388; IC_50_ = 1.38 μM).

In 2012, the Wang group published a communication reporting a new cytotoxic and antifungal metabolite, chaetoglobosin X (**63**) from an endophytic fungus isolated from the leaves of *Curcuma wenyujin* Y.H. Chen et C. Ling, which belongs to the ginger family, collected in Zhejiang Province, Wenzhou, China [45]. Chaetoglobosin X (**63**) exhibited antifungal activity against several plant pathogens, including *Exerohilum turcicum*, *F. oxysporum* f. sp. *Cucumerinim*, *Curvularia lunata* (MIC; 3.125 μg/mL), as well as *F. graminearum* and *F. moniliforme* (MIC; 6.25 μg/mL). Other chaetoglobosins have been reported from the mangrove endophytic fungus *P. chrysogenum* V11 isolated from the vein of the medicinal plant *Myoporum bonitioides* A. Gray in the Leizhou Penninsula in China [46]. These included penochalasin I (**64**), containing an unprecedented six-membered fused ring system, and penochalasin J (**65**). Penochalasin I (**65**) exhibited cytotoxic activity against gastric (SGC-7901) and breast cancer cell lines (MDA-MB-435) with IC_50_ values below 10 μM. Penochalasin J (**65**) exhibited antifungal activity against the plant pathogen *Colletotrichum gloeosporioides* with an MIC of 25.08 μM, which was more active than the known antifungal agent carbendazim. Other antifungal agents have been reported from endophytic fungi from this plant. These agents can be found in the following reports by Li et al. [47,48,49].

## 5. Preclinical and Clinical Trials of Mangrove Endophytic-Sourced Compounds

Secondary metabolites isolated from mangrove fungi have entered preclinical and clinical trials. One metabolite was isolated along with a number of cytotoxic anthracenedione derivatives from the mangrove endophytic fungus *Halorosellina* sp. no 1403 collected from the South China Sea. This compound, the anthroquinone SZ-685C (**66**), was reported by the She group and demonstrated in vitro and in vivo cytotoxic activity [50]. This agent is structurally similar to the clinically used anticancer drug epiadriamycin and exhibited cytotoxic activity against human breast cancer (MCF-7, IC_50_ 7.5 μM; MDA-MB-435, IC_50_ 3.0 μM), prostate cancer (PC-3 cell line, IC_50_ 4.1 μM), glioblastoma (LN-444, IC_50_ 7.8 μM), and hepatoma (Hep-3B, IC_50_ = 3.2 μM; Huh-7, IC_50_ = 9.6 μM) cell lines. Importantly, SZ-685C (**66**) exhibited low cytotoxic activity against healthy epithelial cells [50], and increased sensitivity towards the invasive and estrogen-independent breast cancer cell lines, which are more resistant to anti-estrogen therapy. Thus, SZ-685C (**66**) may be a promising treatment for adriamycin-resistant breast cancer, and a patent was filed in 2010 [51]. Xenograft studies in which mice inoculated with MDA-MB-435 cells were dosed with 50 mg/kg of SZ-685C (**66**) showed a significant decrease of 61% in tumor volume after 35 days of drug administration. SZ-685C (**66**) was later found to selectively induce apoptosis in malignant cells in vitro and in vivo via the inhibition of the Akt pathway, which helps cancer cells survive apoptosis [52].

Additional in vitro assays and mechanistic studies have been published on SZ-685C (**66**). In 2013, Chen and coworkers reported inhibition of pituitary adenoma, specifically rat prolactinoma (MMQ) cells, and normal rat pituitary cells (RPCs) with IC_50_ values of 13.2 mM and 49.1 mM, respectively [53]. The apoptotic activity of SZ-685C in the pituitary adenoma cells resulted from the downregulation of miR-200c, a microRNA implicated in regulating tumorigenesis and commonly upregulated in various tumors. It also inhibited MMQ cells, lowering their miR-200c levels in a dose-dependent manner and exhibited less toxicity toward RPCs. SZ-685C (**66**) was also reported to inhibit human nasopharyngeal carcinoma (CNE2; IC_50_ = 8.97 μM) and its radioresistant analog (CNE2R; IC_50_ =8.94 μM) cell lines after only 72 h of treatment [54]. In 2015, SZ-685C (**66**) was also reported to exhibit cytotoxic activity against non-functioning pituitary adenoma, a benign growth, by upregulating caspase-3 and phosphate and tensin (PTEN) homolog and decreasing Akt expression levels [55]. The lack of selectivity between CNE2 and CNE2R is notable, as radioresistance is a major challenge for naso-pharyngeal carcinoma patients and has been linked to the activation of several pro-apoptotic signaling pathways. Significant decreases in miR-205 expression levels were observed in CNE2R cells incubated with SZ-685C (**66**), inactivating the miR-205-PTEN-Akt and Stat3-Jab1-p27 pathways. Thus, SZ-685C (**66**) could also be a promising treatment for nasopharyngeal carcinoma patients. To date, there have been no additional reports on (pre)-clinical studies on this compound.

## 6. Plinabulin Clinical Trials

Aside from SZ-685C (**66**), the only other marine-derived fungal metabolite in the clinical trials pipeline is the cytotoxic agent plinabulin (NPI-2358; **67**), a synthetic derivative of the diketopiperazine halimide (**68**). Halimide (**68**) is produced by *Aspergillus* sp. CNC-139 isolated from the green alga *Halimeda copiosa* collected from the coast of the Philippine Islands as part of studies funded under an NIH Cooperative grant system known as the NCDDG (National Cooperative Drug Discovery Group). These grants involved both academia and the pharmaceutical industry, with in this case, NIH/NCI involvement. The NCDDG consortium that discovered halimide (**68**) was led by the Fenical group at the Scripps Oceanographic Institution in La Jolla California, with a report covering their initial results published in 2000 [56]. Fenical and Jensen set up the small company *Nereus* to further develop agents from the NCDDG and other microbial sources. Some of the earlier work that led to the identification of plinabulin (**67**) can be found in the 2006 review by Gullo et al. [57]. In combination with docetaxel, plinabulin (**67**) has been used to treat non-small-cell lung cancer patients in Phase III clinical trials, where it had extended long-term survival and reduced neutropenia side effects [58]. 

**Figure 5:**
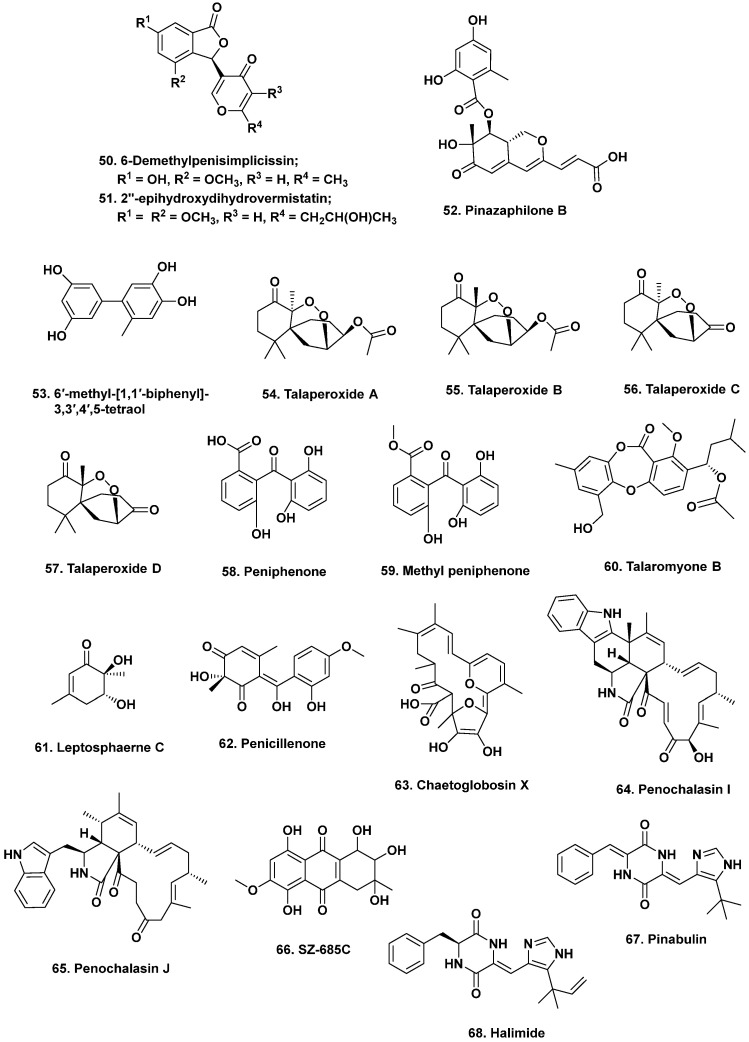
Biologically Active Agents from Mangroves and Other Compounds in Clinical Trials (Structures **50** to **68**).

BeyondSpringPharma who took over the compound following the dissolution of Nereus just (01DEC2021) received a complete response letter from the US FDA, requesting a second Phase III clinical trial for plinabulin (**67**). No comparable response from the China National Medical Products Administration has yet been published. Plinabulin (**67**, Figure 5) is also in Phase I and II trials to treat relapsed small-cell lung cancer patients and non-small-cell lung cancer patients with chemotherapy-induced neutropenia, respectively. 

## 7. Bioactive Compounds from Fungi Isolated from Marine Vertebrates and Selected Invertebrates

In addition to mangrove plants, marine fungi have mutualistic interactions with other organisms, including marine vertebrates, animals with backbones including fish, amphibians, mammals, reptiles, and birds. These animals have commensal, competitive, and predatory interactions with each other. For example, mammals find food by following birds and birds take advantage of the herding efforts of predatory fishes and mammals for food. Furthermore, these animals are active and mobile, covering wide distances to avoid competitors, reducing the rate of evolutionary divergence. Yet, their mobility also provides opportunities to obtain nutrients from different locations, increasing the diversity of their microbiomes.

From initial sequencing studies, the microbiomes of some mammals and marine vertebrates were found, as expected, to have gut microbiota diversity across species [59,60,61,62]. Several fish microbiomes have been sequenced and found to be unique environmental niches for the microbial production of new bioactive molecules [63]. While fish are the most dominant marine vertebrates, only a small number of natural products have so far been reported from these organisms. Fumiquinazolines A–G (**69**–**75**) were produced by the fungus *A. fumigatus* isolated from the gastrointestinal tract of the Japanese saltwater fish *Pseudolabrus japonicas* [64,65]. These peptidyl alkaloids have variable degrees of oxygenation, methylation, and substitution on their indole moieties and all exhibited cytotoxic activity against the murine lymphocytic leukemia (P388) cells with ED_50_ values of 6.1, 16.0, 14.6, 17.7, 52.0, 13.5 and 13.8 μg/mL, respectively.

**Figure 6:**
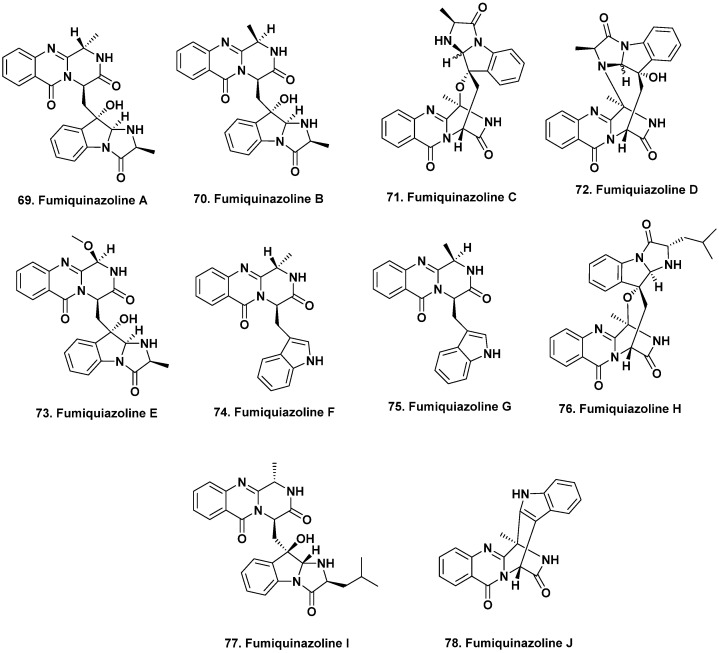
Bioactive Fumiquinazolines from Vertebrates and Invertebrates (Structures **69** to **78**).

This next section might seem out of place, but we have put it here due to the similarity in the structures that were isolated, as this demonstrates that the “nominal host” is perhaps not the controlling factor. The structurally related fumiquinazolines H–L were isolated from fermentation of an *Acremonium* sp. obtained from the *non-vertebrate* marine tunicate *Ecteinascidia turbinata* [66], as well as other marine-derived *A. fumigatus* and endophytic *Scopulariopsis* sp. isolated from *non-vertebrate* gorgonians [67]. Some of these fumiquinazolines inhibited the proliferation of mouse CDC2-mutant (tsFT210) cells, including compounds **69**, **71**, and **74** from the fish-related fungus and fumiquinazoline J (**78**, Figure 6) from the tunicate, while fumiquinazolines H–I (**76**, **77**) exhibited antifungal activity. The 2019 review by Resende et al. [68] is an excellent discussion on the fumiquinazolines, covering their source(s), chemical and biological activities. This review along with the 2020 article in Marine Drugs by Han et al. [69] should be consulted for current information on this class of fungal metabolites and relatives, including genomic aspects underlying their production.

Due to the duplication of structures and names related to fumiquinazolines published from different sources, with approximately 80 mentioned in the Resende et al. review [68], we have used the structures for fumiquinazoline A to J from that source, rather than those first reported in 1992 [64].

## 8. Further Piscine-Sourced Agents

A single fungal strain, *Chaetomium globosum* OUPS-T106B-6 isolated from the marine fish *Mugil cephalus* collected in Katsuura Bay, Japan led to the discovery of several cytotoxic polyketides. Two groups led by Tanaka [70,71], reported six new cytotoxic azaphilones, the chaetomugilins A–F (**79**–**84**), that differed in their degrees of hydroxylation and methylation. All compounds exhibited cytotoxic activity against murine leukemia (P388) and human promyelocytic leukemia (HL-60) cell lines with IC_50_ values ranging from 1.3 to 18.7 μM. The bioactivity of chaetomugilins C (**81**) and F (**84**) indicate that the hydroxyl and methoxy groups at C-12 and C-3′ contribute to their cytotoxicity. Furthermore, chaetomugilins A (**79**), C (**81**), and F (**84**) exhibited selective cytotoxic activity against 39 human cancer cell lines, implying that their modes of action were different from any other anticancer drug developed at the time of publication. Chaetomugilins G–H (**85**–**86**), azaphilones with hydrolyzed lactone rings, were also reported by the authors from the same fungal strain [72]. Both compounds exhibited growth inhibitory activity against murine lymphocytic leukemia (P388 and L1210) as well as human promyelocytic leukemia (HL-60) and epidermoid carcinoma (KB) cells with IC_50_ values ranging from reasonably active (10.3 μM) to effectively inactive (137.8 μM) for pure compounds.

**Figure 7:**
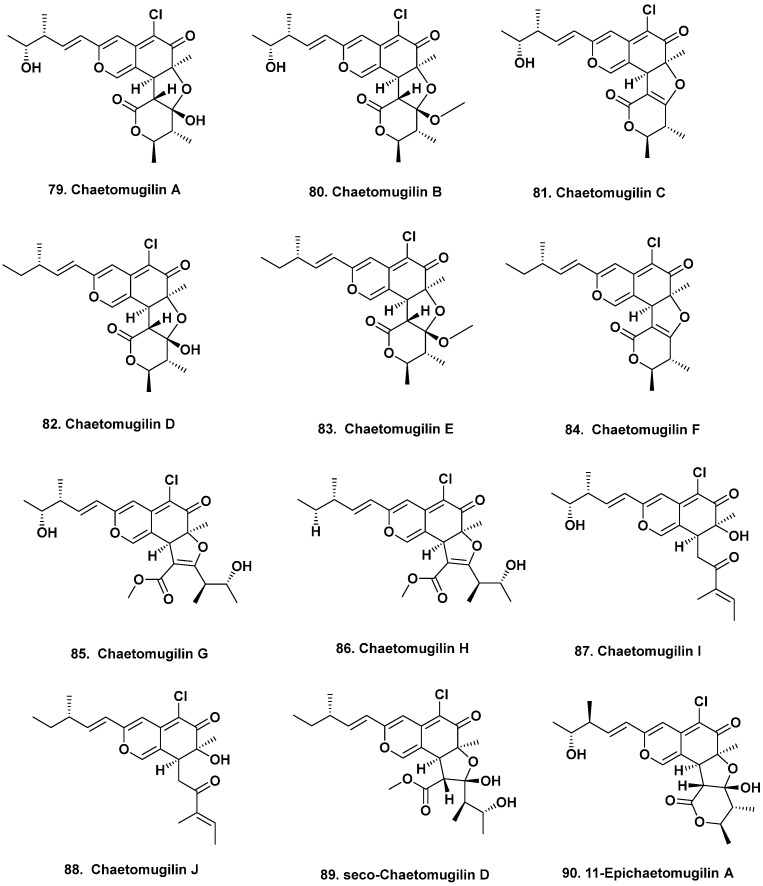
Bioactive Chaetomugulins and Derivatives from Piscine Sources (Structures **79** to **90**).

The same research group later reported chaetomugilins I–O, which had either a five-membered ether ring or various oxygenation and methylation patterns, from the same fungal strain [73]. Chaetomugilin I (**87**) also exhibited select cytotoxic activity against the same 39 human cancer cell lines mentioned above. Chaetomuglin J (**88**) was also recently reported to enhance apoptosis induced by cisplatin in human ovarian cancer cells, increasing their sensitivity to cisplatin by decreasing mitochondrial membrane potentials and increasing reactive oxygen species levels [74]. Derivatives in which the lactone moiety was hydrolyzed, seco-chaetomugilins A and D (**89**), were also found in the culture broth of the same fungal isolate [75]. The lack of the C-12 hydroxyl group in seco-chaetomugilin D possibly contributed to its weak cytotoxicity against the murine leukemias (P388 and L1210) as well as human leukemia and epidermoid carcinoma (KB) cell lines with values of 38.6, 53.6, 47.2, and 47.2 μM, respectively. Another weakly cytotoxic derivative, 11-epichaetomugilin A (**90**, Figure 7), was later reported by the same authors and it too, only exhibited weak cytotoxic inhibitory activity against murine lymphocytic leukemia (P388) and human promyelocytic leukemia (HL-60) cells with IC_50_ values of 88.9 and 66.7 μM, respectively [76]. The chaetomugilin BGC has been characterized and should be further explored for new derivatives, as its structural scaffold appears to selectively target tumor cells and increase their sensitivity to other antitumor drugs [77].

## 9. Bioactive Compounds from Fungi from Marine Invertebrates, Seagrass, Cyanobacteria and Algae

The “nominal sources above” have been a rich source of bioactive fungal metabolites, especially because sponges are the most well-studied environment for bioactive agents and their “actual source(s).” Sponges usually have over 50% of their mass composed of single-celled organisms which are the potential actual sources of the secondary metabolites isolated from the host invertebrate. Several cytotoxic metabolites have been reported from fungal strains isolated from sponges.

A new cyclohexapeptide (similanamide, **91**) was isolated from cultures of the sponge-associated *Aspergillus similanensis* KUFA0013. The strain was isolated from the marine sponge *Rhadbermia* sp. collected from the coral reef of the Similan Islands in Thailand at a depth of 10 m [78]. Similanamide (**91**) exhibited weak activity against breast adenocarcinoma (MCF-7), non-small-cell lung cancer (NCI-H460), and melanoma cell lines with GI_50_ values ranging from 115 to 125 μg/mL [79]. In 2015, Masuda et al. [80] published a revision of the structure confirmed by total synthesis, identifying it as one of the two cyclic peptides reported in 2010 by Kai et al. Both peptides resulted from a cofermentation of the unidentified ascomycete OK-128 with okara, and exhibited paralytic activity against silkworms [81].

Seven new polyketides were isolated from *Alternaria* sp. SCIO41014 collected from the sponge *Callyspongia* sp. collected near Xuwen County, Guangdong Province, China. One of the isolated perylenequinone derivatives, 4,8,10-trihydroxy-1,2,11,12-tetrahydroperylene-3-quinone (also named Altertoxin VII, **92**), exhibited cytotoxic activity against a wide range of cancer cells, including human erythroleukemia (K562), human gastric carcinoma cells (SGC-7901), and hepatocellular carcinoma cells (BEL-7402) with IC_50_ values of 26.58 ± 0.80, 8.57 ± 0.13 and 13.11 ± 0.95 ug/mL, respectively [82].

New sorbicillinoid derivatives have been reported from a fungus isolated from the fresh internal tissue of a sponge collected from the South China Sea. Trichoreeseiones A–B, trichodermolide B, 13-hydroxy-trichodermolide, 24-hydroxy-trichodimerol (**93**), and 15-hydroxy-bisvertinol were isolated from *Trichoderma reesei* (HN-2016-018). Notably, 24-hydroxy-trichodimerol exhibited cytotoxic activity against lung (A549), breast (MCF-7), and liver (HCT-116) cancer cell lines with IC_50_ values of 5.1, 9.5, and 13.7 μM, respectively [83].

**Figure 8:**
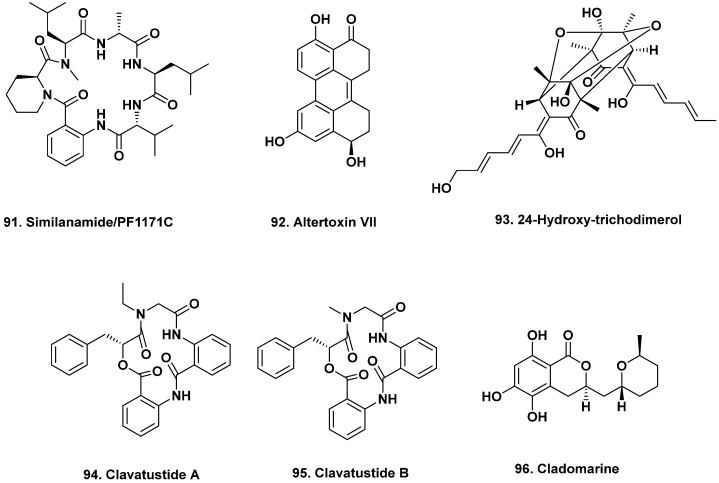
Bioactive Agents from Fungi Isolated from Multiple Sources (Structures **91** to **96**).

Crabs and sea cucumbers have also been a source of bioactive fungal secondary metabolites. The cyclic depsipeptides clavatustides A–B (**94**–**95**) were isolated from *Aspergillus clavatus* C2WU isolated from the crab *Xenograpsus testudinatus* from the hydrothermal vent Kueishantao in Taiwan [84]. Both compounds inhibited HepG2 liver cancer cell growth in the G1/S phase transition. An antimicrobial agent, cladomarine (**96**), was isolated from a sea-cucumber-derived fungus *Penicillium coralligerum* YK-247 collected at a depth of 3064 m on the Sao Paulo Plateau off the coast of Brazil. Cladomarine (**96**, Figure 8) exhibited antimicrobial activity in disc diffusion assays against the pathogens *Saprolegnia parasitica* and *Pythium* sp. sakari1 at a concentration of 10 μg per disc [85]. 

In addition to these specific agents, there are two reviews covering marine invertebrates and their symbiotic microbes, one in 2019 by Lui et al. [86] and the other in 2021 by Marchese et al. [87]. Inspection of these recent reviews will give a broader understanding of the value of these marine sources as leads to novel drug candidates. It should also be emphasized at this point that we have only dealt with fungi that can be grown following isolation from marine sources. It is highly probably that there also exist fungi that are as yet unculturable and whose existence is not yet fully confirmed. Such organisms would be similar to the bacterial species described by the Piel group from 2014 onwards [88,89].

## 10. Bioactive Compounds from Epigenetic Modifications

The sequencing of fungal genomes has revealed that we have underestimated their genetic potential to make natural products, as there are many more genes involved in secondary metabolism than detected gene products. For example, the model soil fungus *A. nidulans* has 28 putative polyketide synthase (PKS) and 24 putative non-ribosomal peptide synthetase (NRPS) gene clusters, demonstrating its capability to produce at least 52 secondary metabolites [90], most of which are silent or cryptic [91].

A variety of techniques have been used to activate these BGCs, including chromatin remodeling, leading to the opening of the cell’s chromatin to enable gene transcription. Chromatic remodeling can occur via DNA methylation and histone modification, which affect the transcription of telomere-proximal BGCs and the levels of corresponding metabolites. Histone proteins undergo a wide array of posttranslational modifications that affect transcription, including methylation, phosphorylation, acetylation, ubiquitination, and sumoylation. As such, chemical epigenetic modifiers, such as Zn^2+^-type histone deacetylase (HDAC) and DNA methyltransferase inhibitors, have been added to fermentation media and successfully stimulated the expression of cryptic BGCs [92].

Several studies have reported the induction of cryptic metabolites by supplementing media with chemical epigenetic modifying agents. The HDAC inhibitor suberohydroxamic acid (SBHA) induced the production of a new biphenyl derivative versiperol A (**97**) in the fermentation of *A. versicolor* MCCC 3A00080, which was isolated from deep-sea sediment (2721 m deep) in the Pacific Ocean [93]. Versiperol A inhibited the growth of *S. aureus* with an MIC value of 8 μg/mL. Other compounds have also been produced by a single strain grown in media containing an HDAC or DNA methyltransferase inhibitor. For example, 5-azacytidine was added to cultures of mangrove-derived *P. variabile* HXQ-H-1 and reported to produce a new fatty acid amide varitatin A (**98**) [94]. This fatty acid amide demonstrated cytotoxicity against colon carcinoma (HCT-116) cells with an IC_50_ value of 2.8 μM. It also inhibited protein tyrosine kinases, such as PDGFR-β and ErbB4 kinases with inhibitory rates of 50% and 40%, respectively, at a concentration of 1 μM. When the same strain was cultivated in the presence of the HDAC inhibitor suberoylanilide hydroxamic acid (SAHA), four new compounds, varilactones A and B, and wortmannilactones M and N were produced [95]. None of these compounds exhibited cytotoxic activity or antiviral activity against the influenza A (H1N1) virus. However, this is a great example of how structural diversity can be accessed in a single strain via the addition of epigenetic modifiers.

Many compounds exhibiting non-cytotoxic or non-infective bioactivities have been reported from deep-sea fungi grown in the presence of DNA or HDAC inhibitors. Readers should consult the work of Wang et al. [96], Beau et al. [97], and Zhang et al. [98] for details of the new fungal metabolites induced by the addition of DNA and HDAC inhibitors.

## 11. OSMAC

One strain–many compounds (OSMAC) is another successful approach used to elicit the production of cryptic metabolites. This is actually a renaming of a very old system used initially by antibiotic researchers in the pharmaceutical industry, but never formally published as such. As an example, one of the authors (DJN) was using this technique as a laboratory assistant in the UK in the late 1950s. Since all industrial researchers knew what was used as media components by colleagues in other pharmaceutical and/or agrichemical houses due to frequent personnel movement between companies, there was no need for any formal paper. This also applied up through the 1980s in industry as well.

The first “formal academic publication” discussing these multiple media techniques was published by Zahner in 1979 though it was based on a lecture in 1977 [99]. In addition, in 1982, a book covering the methods for search and discovery of bioactive microbial products was published by the UK “Society for General Microbiology” that included a relevant article by Zahner, based on his earlier publications, but the essential point was that of the three editors, two were from the UK pharmaceutical industry who were involved in the active search for novel antibiotics, whilst the third editor was at the Microbial Chemistry laboratory at the University of Manchester [100]. This book was not well known outside of UK and EU microbial researchers, but the essential point is that it preceded the “nominal OSMAC” story by 20 years. The current term for this old technique “OSMAC” came from a paper in 2002 by Bode et al. [2], but no attribution was given to Zahner’s earlier published work which was known in German academic circles.

With all microbes, including fungi, the methods involve a systematic modification of cultivation conditions to trigger cryptic gene clusters, as microbial metabolomics change in response to stress stimuli (e.g., pH, temperature, and the addition of metals, antibiotics, and flavonoids) [101]. In addition to varying cultivation parameters, fermentation media can also be supplemented with precursors and enzyme inhibitors [102]. This approach has successfully induced several compounds in marine fungal cultures, especially in *Spicaria elegans* KLA03 CCTCC M 205049. For example, Lin and coworkers reported the production of new cytochalasins, aspochalasins (M–Q), and spicochalasin A (**99**) when *S. elegans* from marine sediment in Jiaozhou Bay, China was cultivated on 2% starch as the sole carbon source [103]. Spicochalasin A (**99**) with an unusual pentacyclic system, exhibited cytotoxic activity against human leukemic (HL-60) cells with an IC_50_ value of 19.9 μM. A revised structure was published by a different group under the name flavichalasine D in 2021, with structure (**99**) being the revised version [104]. Interestingly, the same gene cluster that produced spicochalasin A produced nine new cytochalasins, when grown on glucose, demonstrating the effects primary metabolism can have on secondary metabolism [105,106].

Simply varying salinity has induced the production of structurally diverse fungal metabolites. The endophytic *Nigrospora* sp. MA75 isolated from the marine semi-mangrove plant *Pongamia pinnata* was found to produce a mixed polyketide-terpenoid, 2,3-didehydro-19α-hydroxy-14-epicochlioquinone B (**100**), when cultivated in media containing 3.5% NaCl [107]. Two new griseofulvin derivatives, 6-*O*-desmethyldechlorogriseofulvin and 6′-hydroxy-griseofulvin, were also isolated when the same strain was cultured on solid rice medium. In addition to antibacterial activity, compound **100** exhibited cytotoxic activities against human breast cancer (MCF-7), pancreatic cancer (SW1990), and hepatocellular cancer (SMMC7721) cell lines with IC_50_ values of 4, 5, and 7 μg/mL, respectively [107].

When *Aspergillus* sp. SCSIO F063 isolated from a deep-sea sediment in the South China Sea was cultured in potato dextrose broth supplemented with 3% sea salt, seven new chlorinated anthraquinones were produced. (1′*S*)-7-Chloroaverantin, (1′*S*)-6-*O*-methyl-7-chloroaverantin (**101**), (1′*S*)-1′-*O*-methyl-7-chloroaverantin, (1′*S*)-6,1′-*O*,*O*-dimethyl-7-chloroaverantin, (1′*S*)-7-chloroaverantin-1′-butyl ether, 7-chloroaverythrin, and 6-*O*-methyl-7-chloroaverythrin are anthraquinones with a chlorine atom at C-7 as well as different methoxy, *n*-butyl, and unsaturated alkyl chain groups [108]. Two new brominated compounds were produced when the culture broth of the same strain was supplemented with 3% sodium bromide. Of all of the new metabolites produced, only 6-*O*-methyl-7-chloroaverantin (**101**) exhibited strong cytotoxic activity against human glioblastoma (SF-268), breast cancer (MCF-7), and lung cancer (NCI-H460) cell lines with IC_50_ values of 7.11, 6.64, and 7.42 μM, respectively.

When grown in media containing 10% saline, *S. elegans* KLA03 isolated from a marine sediment in Jiaozhou Bay, China produced (2*E*,2′*Z*)-3,3′-(6,6′-dihydroxybiphenyl-3,3′-diyl)diacrylic acid (**102**) [109]. Compound **102** exhibited antibacterial activity against *Pseudomonas aeruginosa* and *E. coli* with MIC values of 0.038 mM and 0.767 mM, respectively. When Luan et al. grew the same strain in a mannitol-based medium using ammonium chloride as the nitrogen source, it produced a small amount of a rare, unstable, highly oxygenated spiro[isobenzofuran-1,3′-isochroman] ring system, eleganketal A (**103**), which could not be evaluated for bioactivity, but the structure was confirmed by synthesis of the permethylated derivative [110].

Thus, simply modifying salinity and/or the halide ion can produce a variety of bioactive agents hopefully providing enough material that can be isolated for biological evaluation, or alternatively the agent might be synthesized. As examples of varying the halide, a new radical scavenging brominated isocoumarin, (*R*)-(–)-5-bromomellein (**104**) was also produced once sodium bromide was added to the fermentation broth of the fungus *A. ochraceus* isolated from the marine red alga *Chondria crassicualis* collected in Korea [111]. Compound (**104**) exhibited radical scavenging activity against 1,1-diphenyl-2-picrylhydrazyl radical 2,2-diphenyl-1-(2,4,6-trinitrophenyl) hydrazyl (DPPH) with an IC_50_ value of 24 μM, which was comparable to the L-ascorbic acid positive control (IC_50_ = 20 μM).

Supplementing the fermentation broth with amino acids can produce amino acid derivatives, again a technique known from many years earlier as “precursor-directed biosynthesis”. Wang and coworkers used this strategy to obtain new cytochalasins from an *S. elegans* KLA03 strain. Three new cytochalasins, cytochalasins Z_21_ (**105**), Z_22_ (**106**), and Z_23_ were produced when cultures were supplemented with L- and D-tryptophan [112]. Cytochalasins are derived from acetate, a methionine-derived octa- or nonaketide chain, and an amino acid. Notably, cytochalasins Z_21_ (**105**) and Z_22_ (**106**) exhibited cytotoxic activity against human lung adenocarcinoma (A-549) with IC_50_ values of 8.2 and 20.0 μM, respectively.

The addition of L-tryptophan and L-phenylalanine to the fermentation broth of *Dichotomomyces cejpii* F31-1 isolated from the soft coral *Lobophytum crassum* collected in the South China Sea has also led to the production of several new compounds [113]. Four amides, dichotomocejs A (**107**) to D, one polyketide dichocetide A, and two diketopiperazines dichocerazines A–B were produced. Dichotomocej A (**107**) exhibited weak activity against the human rhabdomyosarcoma cell line RD with an IC_50_ value of 39.1 μM. The other dichotomocejs were not evaluated but it would be interesting to see how the differing structural substitutions might have altered their biological activity.

**Figure 9:**
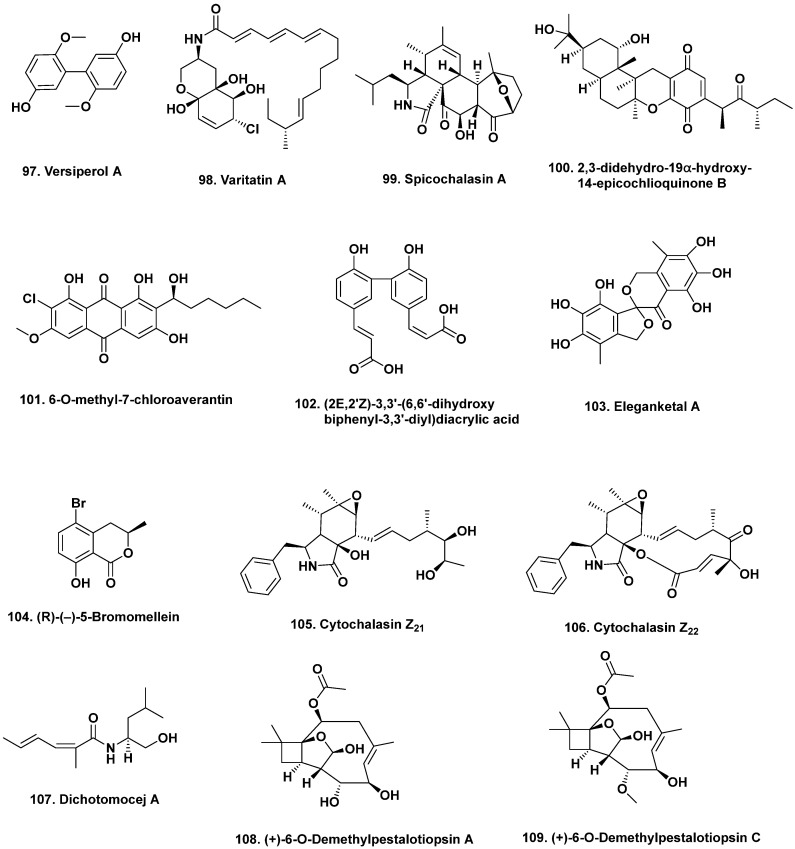
Modification of Media Components/Fermentation Conditions I (Structures **97**–**109**).

Metals have been added to fermentation broths to induce the production of new metabolites. Free metals can function as a “chemical switch” and upregulate biosynthetic pathways or they can be used in either dissimilatory or assimilatory reactions. Some organisms, such as hydrothermal vent microorganisms, are particularly sensitive to changes in the concentrations of available metals, such as copper, and produce new metabolites, many of which have not been assessed for bioactivity [114,115,116]. Wang et al. [117], reported that *Ascotricha* sp. ZJ-M-5 isolated from coastal mud in Fenghua County, Zhejiang Province, China produced new caryophyllene derivatives, (+)-6-*O*-demethylpestalotiopsin A (**108**), (+)-6-*O*-demethyl-pestalotiopsin C (**109**), and (-)-6-*O*-demethylpestalotiopsin B, when cultivated in Czapek Dox broth with or without Mg^2+^. This is the first time caryophyllene derivatives have been detected in *Ascotricha* cultures. (+)-6-*O*-Demethylpestalotiopsin A (**108**) and (+)-6-*O*-demethyl-pestalotiopsin C (**109**, Figure 9) exhibited growth inhibitory activity against human promyelocytic leukemia (HL-60; GI_50_ = 6.9 and 8.5 μM) and human chronic myelogenous leukemia (K562; GI_50_ = 10.1 and 12.3 μM) cell lines.

Switching from liquid to solid media can also trigger the production of new metabolites. For example, nine new C_9_ polyketides were produced by the hydrothermal vent-derived *Aspergillus* sp. 16-02-1 (Lau Basin hydrothermal vent, southwest Pacific at 2255 m and 114 °C) when grown on rice [118], as opposed to a liquid medium, which only produced known compounds [119]. When re-fermented on solid substrate, aspiketolactonol (**110**), aspilactonols A–F (though E and F are an unseparated mixture of epimers) (**111**–**115**), aspyronol (**116**), epiaspinonediol (**117**), and five new ambuic acid derivatives were produced. Compounds **110**–**115** are α,β-unsaturated *γ*-lactones with varying degrees of oxygenation, whereas compound **116** is an α,β-unsaturated *δ*-lactone ring and compound **117** is a branched, acyclic C_8_ carbon chain with a conjugated diene moiety. All compounds (**110**–**117**) inhibited human leukemia and gastric cancer cell lines (K562, HL-60, and BGC-823 cells) to varying degrees. Aspyronol (**116**) and epiaspinonediol (**117**) were more potent and selective cytotoxic agents on human leukemia (K562 and HL-60) cell lines. 

When Guo and coworkers switched from growing a deep-sea-derived *Penicillium* sp. F23-2 (from a Chinese deep ocean sediment at 5080 m) in complex liquid media to a rice-based solid medium [120], penicyclones A–E (**118**–**122**) were produced. This strain was already reported to produce meleagrins, roquefortines, and terpenoids in a potato-based medium under static conditions and sorbicillinoids in peptone yeast glucose broth medium with agitation. Penicyclones A–E (**118**–**122**) differ in their degrees of oxygenation, unsaturation, and alkyl chain length. Yet, all compounds exhibited antimicrobial activity against *S. aureus* with MIC values ranging from 0.3 to 1.0 μg/mL.

**Figure 10:**
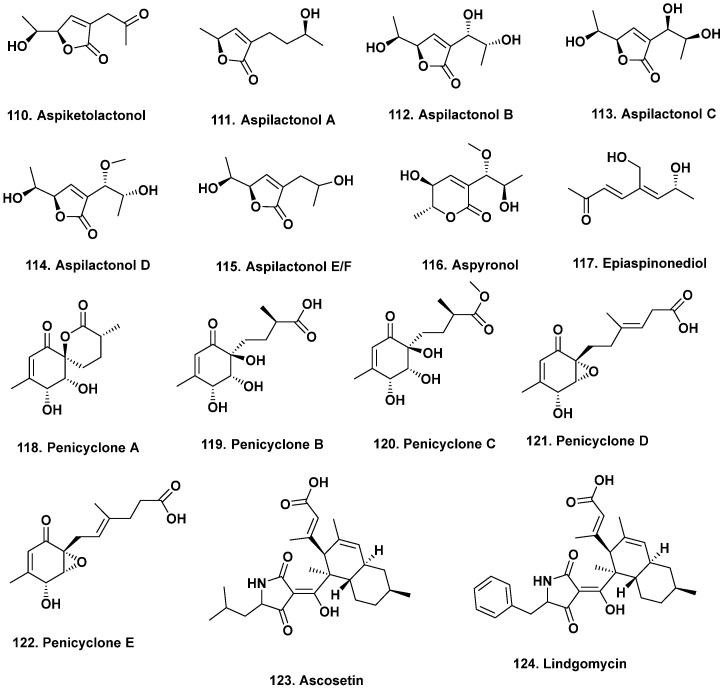
Modification of Media Components / Fermentation Conditions II (Structures **110**–**124**).

Simple changes in fermentation vessels can also improve yields and reduce processing times. For example, the yields of the antibiotics ascosetin (**123**) and lindgomycin (**124**, Figure 10) from an Arctic-sourced *Lindgomycetaceae* isolated from the sponge *Halichondria panicea*, improved by a factor of 100 once transferred from an Erlenmeyer flask to a stirred tank reactor [121].

Environmental cues, such as temperature, can trigger the expression of silent gene clusters. Liu and coworkers reported five new polyketides produced from *P. raistrickii* JH-18 isolated from coastal marine soil from Bohai Bay, China once the fermentation temperature was lowered from 28 °C to 15 °C [122]. The change in the temperature significantly altered the *P. raistrickii* JH-18 metabolome and new raistrickione diastereomers, raistrickiones A–C (**125**–**127**), and analogs, raistrickiones D and E (**128**–**129**), were produced. Raistrickiones A–E exhibited radical scavenging activity against DPPH with IC_50_ values of 32, 38, 40, 49, and 42 μM, respectively. The IC_50_ value of the ascorbic acid positive control was 17 μM. It would be interesting to know the optimal growth temperature of this *Penicillium* strain to understand why these metabolites are produced.

Supplementing culture media with enzyme inhibitors has also led to the production of new bioactive compounds. For example, based on several species of *Phomopsis* and *Chaetomium* sp. producing well-known actin depolymerizers (i.e., chaetoglobosins), the Crews group added an actin inhibitor to induce the production of unprecedented chaetoglobosins. Three new chaetoglobosins with various levels of oxygenation, chaetoglobosin-510, -540, and -542 (**130**), were produced when the actin inhibitor jasplakinolide (*aka* jaspamide) was added to cultures of *Phomopsis asparagi* isolated from the sponge *Rhaphidophlus juniperina* collected from the US Virgin Islands [123]. Chaetoglobosin-542 (**131**) exhibited antimicrofilament activity at 1 μg/mL as well as cytotoxic activity against murine colon and leukemia cell lines but was also toxic toward murine normal bone marrow (CFU-GM). 

Other enzyme inhibitors, such as the P-450 dependent monooxygenase inhibitor metyrapone, have been used to induce the production of new metabolites [124]. For more information on the application of these well-known principles to marine fungi, see the reviews by Reen et al. [125] and Romano et al. [126]. 

## 12. Optimization of Growth Conditions and Strains for Large-Scale Production

A common problem in finding new bioactive fungal metabolites is not having enough isolated material for biological evaluation. However, metabolite production can be further optimized by modifying growth conditions. For example, altering the salinity has also improved the yields of the antibiotics obioninene (**131**) as well as corollosporine (**132**) and derivatives (in the presence of laccase treatment) produced by driftwood-derived *Leptosphaeria oraemaris* [127], and *Corollospora maritime* [128,129]. Time is also an important factor that has been found to influence the accumulation or depletion of metabolites, with several studies reporting the impact of time on activating hidden metabolomes [130].

Several variables can be modified at once to optimize the yields of isolated fungal metabolites. For example, in optimized medium, cultivation time of three days, and 28 °C growth temperature, the yield of the antibacterial, lipodepsipeptide 15G256γ (**133**) produced by mangrove-derived *Hypoxylon oceanicum* LL-15G256 (Shenzen, China) increased from ~50 to 400 mg/L [131]. In addition, simple modifications in pH and growth time were also shown to be important for producing optimal amounts of the antibacterial exophilin A (**134**), which was produced by *Exophilia pisciphila* isolated from the sponge *Mycale adhaerans* [132]. Furthermore, spore inoculation, low agitation (50 rpm) and pH (4.5), an aeration rate of 0.33 vvm, and a 2-fold amount of maltose and glucose on the sixth day of growth in cultures of *A. terreus* PF-26 (isolated from the sponge *Phakellia fusca*) grown in a stirred 5-L reactor led to a 1.65-fold increase in (+)-terrein, (**135**) a cytotoxic antibiotic to 2.68 g/L) [133]. While these modifications have been successful, using the “one-factor-at-a-time” approach can be time-consuming and does not reveal the interactions of various parameters.

Controlled and targeted methods, involving statistical analyses, can modify multiple variables simultaneously. Alternative methodologies, including orthogonal array designs, Plackett–Burman, and response surface methodologies (RSM), have been developed to optimize product yields by examining the effects of multiple variables in parallel in a minimal set of experiments.

Genichi Taguchi developed the orthogonal array design, a statistical method that determines how different parameters affect product yields in the smallest number of experiments, without having to explore all possible combinations [134]. Though somewhat “dated” this strategy provides the significant and optimal contributions of each variable.

The Plackett–Burman experimental design, though originally published in 1946, measures the dependence of a measured quantity on various complex media components in a way that minimizes the variance of these dependencies using a limited number of experiments [135]. This technique was utilized extensively in the earlier days of antibiotic production when companies wished to reduce the sizes of their production fermentors.

RSM is another sequential technique that involves designing experiments, mathematical models, and statistical analyses to explore relationships between a response variable and a set of design variables [136]. This approach determines influential factors and how they interact with each other, revealing the role of each component in the optimization process.

Orthogonal array designs, Plackett–Burman designs, and RSM were all used to increase the yield of the antineoplastic compound aspergiolide A (**136**) from the marine-derived *A. glaucus* HB1-19 by 4.22-fold (71.2 mg/L) [137,138]. RSM was also used to optimize the antineoplastic, antibiotic penicilazaphilone C (**137**) produced by the marine-derived fungus *P. sclerotiorum* M-22, first reported by Zhou et al. in 2016 [139], and then optimized increasing the yield by 1.344-fold [140].

DNA modifications and genetic engineering have also been used to coerce fungal strains into producing higher yields. For example, ultraviolet radiation was used to mutate the DNA of *Scopulariopsis brevicaulis* LF580 to optimize the yield of the sponge-derived scopularide A (**138**) [141]. This cyclic peptide whose structure was revised in 2016 [142], exhibited cytotoxic activity against pancreatic and colon tumor cells [143,144]. In this optimization work, a faster-growing UV-mutated strain (LF580-M26) that rapidly produced more scopularide A was identified. The LF580-M26 strain showed differences in pellet formation, which is relevant as proteomics revealed the wild-type strain’s limited nutrient availability due to its robust pellet formation.

**Figure 11:**
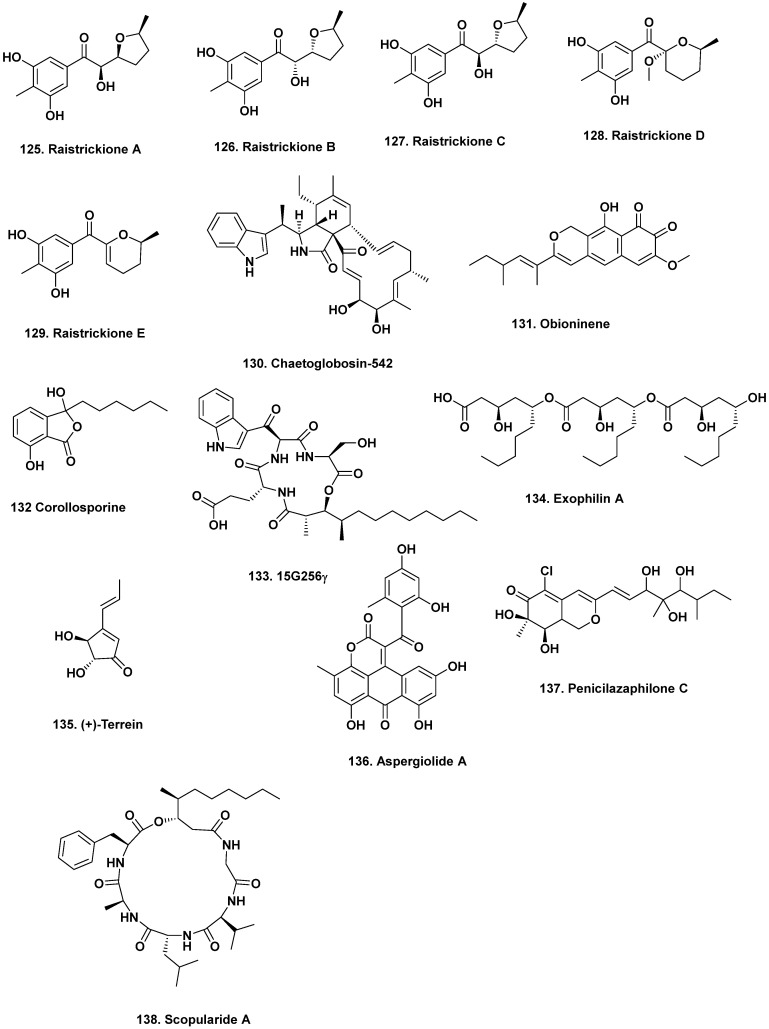
Modification of Media Components / Fermentation Conditions III (Structures **125**–**138**).

Genetic engineering has also improved metabolite yields by (in)activating genomic segments via the manipulation of regulators or chromatin-modifying enzymes. Scopularide A (**138**, Figure 11) was optimally produced by *S*. *brevicaulis* LF580 once its biosynthetic gene cluster was identified [145]. The following papers and reviews give more information on producing bioactive fungal metabolites via genetic engineering [91,146,147].

## 13. Materials Produced by Both Terrestrial and Marine Isolates

**Figure 12:**
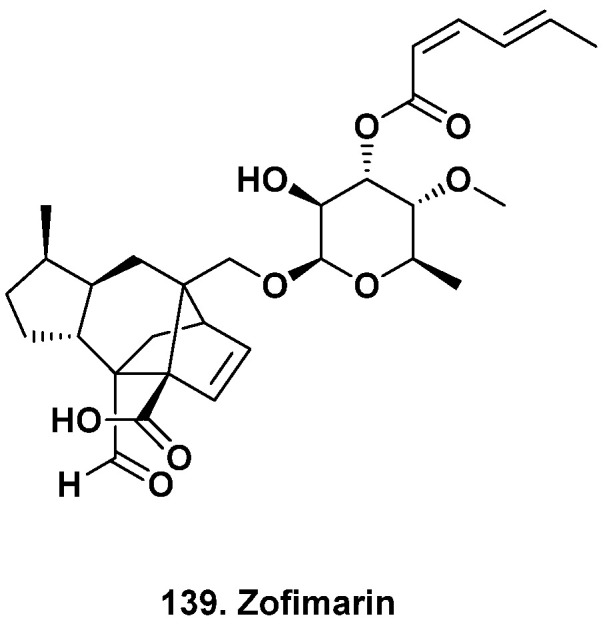
Zofimarin, a Fungal Metabolite from Land and Sea (Structure **139**).

Let us also not forget that several “marine” natural products are also produced by terrestrial endophytic fungi, some of which are more amenable to optimizing product yields. For example, the antifungal zofimarin (**139**, Figure 12) was originally isolated from the marine fungus *Zopfiella marina* SANK21274 but was later isolated from the fermentation broth of *Xylaria* sp. Acra L38 from the Thai medicinal plant *Aquilaria crassna* [148]. Chaichanan and coworkers grew *Xylaria* sp. Acra L38 in sucrose, maltose, glucose, and sodium nitrate and increased the zofimarin yield 8-fold compared to when it was grown in Czapek yeast extract. Many of the natural products discussed here, especially those that are mangrove-derived, may also be produced by terrestrial fungi, which could be explored for greater product supplies.

## 14. Conclusions

While we will continue to see an upward trend in the number of bioactive metabolites from marine fungi with increased marine exploration, several underrated strategies should be considered moving forward. Marine fungi are typically grown using the same cultivation methods used with terrestrial fungi, which rarely reflect the metabolic demands of marine fungi. Thus, increased collaborations between natural product chemists and mycologists, as described by Reich and Labes in a paper in Marine Genomics in December 2017 [149], would aid in ‘unlocking’ more fungal metabolomes from the sea. Most fungal strains discussed in this review belong to cosmopolitan genera, such as *Penicillium* and *Aspergillus*; however, mycologists have strategies for isolating and cultivating unique marine fungal isolates.

Advances in culturing, screening, and culture-independent techniques will also allow us to access more metabolites from extremophilic fungi, resulting in increased chemical diversity and bioactivity [150]. The cocultivation of marine fungi with other organisms, including with fungi should be considered, as exemplified by the Stierle group at the University of Montana in a paper a year earlier [151]. This fermentation method takes advantage of symbiotic or competitive growth conditions to trigger secondary metabolite production via upregulating silent genes. Regarding screening and culture-independent approaches, readers should consult the recent paper by Nie et al. [152], the reviews by Lage et al. [153] and Wang et al. [154], to see how these research areas are also providing investigators with tools to access bioactive fungal metabolites. 

Lastly, more research groups from China are publishing on bioactive metabolites from marine microorganisms with a focus on fungi [35]. This trend might represent a shift with reports being published in international journals instead of Chinese journals, but we expect to see more publications from this region, especially on compounds produced by mangrove endophytic fungi. For more bioactive fungal metabolites from the sea, we recommend reading the special 2020 issues from Marine Drugs, “Bioactive Compounds from Marine Sediment Derived Fungi” and “Natural Products from Marine Fungi” which are freely available on the Marine Drugs website.
